# Observational study on effectiveness and safety of integrative Korean medicine treatment for inpatients with sciatica due to lumbar intervertebral disc herniation

**DOI:** 10.1097/MD.0000000000020083

**Published:** 2020-05-22

**Authors:** Yoon Jae Lee, Jongho Kim, Me-riong Kim, Joowon Kim, Min-Young Kim, Hyun-Woo Cho, Sook-Hyun Lee, Inhyuk Ha

**Affiliations:** a Jaseng Spine and Joint Research Institute, Jaseng Medical Foundation; b Jaseng Hospital of Korean Medicine, Seoul; c Bucheon Jaseng Hospital of Korean Medicine, Gyeonggi-do; d Daejeon Jaseng Hospital of Korean Medicine, Daejeon; e Haeundae Jaseng Hospital of Korean Medicine, Busan, Republic of Korea.

**Keywords:** complementary therapies, intervertebral disc displacement, low back pain, lumber intervertebral disc herniation, prospective study, protocol, registry

## Abstract

We developed a protocol for a prospective registry to prove the effectiveness and safety of integrative Korean medicine treatment for inpatients with sciatica due to lumbar intervertebral disc herniation. We plan on recruiting 1000 inpatients receiving integrative Korean medicine treatment for lumbar intervertebral disc herniation at four spine specialized Korean medical hospitals. Patients enrolled in the registry will be evaluated at the time of hospitalization, 2 weeks after hospitalization, at discharge, and 6 months after hospitalization on predefined outcome variables such as intensity of back and leg pain, Oswestry Disability Index, quality of life, Patient Global Impression of Change, and adverse effects. The protocol of this study was registered in CRIS (KCT0003709) and Clinical trial gov (NCT03750591). This study is significant in that it cannot only be a basis for safety-related evidence of complementary alternative medicine, which has been lacking, but it also gives clear evidence on the effectiveness and validity of treatment effects such as accompaniment of stenosis, sex, age, and type of disc herniation.

## 1 Introduction

Lumbar intervertebral disc herniation (LDH) is characterized by pain, weakness, or numbness in myotomal or dermatomal distribution due to localized displacement of disc material.^[1]^ Low back pain and sciatica, the most common symptom of LDH, are limitations in daily life. From the Global Burden of Disease 2010 study,^[2]^ low back pain was the highest disease burden in the Years Lived with disability; in other words, LDH is responsible for most disease burdens worldwide. Conservative treatments and surgical treatments have been suggested^[1]^ as treatment for LDH, but there are limitations as patients might complain of Failed Back Surgery Syndrome (FBSS) after surgery.^[3]^ Therefore, nonsurgical integrative treatment using Complementary Alternative Medicine (CAM) is being used in Korea.

In Korea, a combination treatment using acupuncture, herbal medicine, chuna manual therapy, and pharmacopuncture is being used on LDH patients. Particularly, inpatient treatment is often given to LDH patients who suffer from severe pain that affects their daily lives. Jaseng Hospital of Korean Medicine, a spine specialized Korean medical hospital designated by the Korean Ministry of Health and Welfare, treats many LDH patients. In the past, we reported on the effectiveness of inpatient treatment for LDH patients in a single center, and long-term follow-up studies confirmed that the effect was maintained.^[4,5]^ Based on these previous studies, we tried to establish a multicenter registry to systematically prove the effectiveness and safety of integrative Korean medicine treatment for inpatients with LDH by obtaining additional clinical indicators and safety data.

There are several reports on the effects of CAM treatment on musculoskeletal disorders in clinical studies.^[6,7]^ However, in the systematic literature review, treatment protocols differ for each study, many are of small size, and there are few related studies to draw clear conclusions.^[8,9]^ Particularly, safety-related studies may require more subjects, but because CAM treatment studies thus far have often involved a small number of patients, prospective safety data in large number of patients are necessary.

The registry can recruit a large number of patients, has a high degree of generalization of findings, and has an ability to detect rare symptoms such as side effects. In the musculoskeletal disorders, various registries have been established^[10,11]^ and have been helpful in demonstrating its safety and efficacy, but there is no registry related to integrative Korean medicine treatment. Therefore, as part of the Joint and Spine Pain Integrative Evaluation registry (J-SPINE) project, we want to establish a registry for LDH. We aim to build a prospective large-scale registry study based on well-established research designs to confirm the clinical effectiveness and safety of Korean medicine treatment in LDH patients.

## 2 Method

### 2.1 Design

#### 2.1.1 Protocol overview

This study is a prospective observational study having a registry and no control group. All treatments during hospitalization will be recorded with no restrictions. We plan on recruiting 1000 patients admitted at 4 spine specialized Korean medical hospitals. Patients enrolled in the registry will be evaluated on predefined outcome variables (pain, quality of life, disability, and physical examination) at the time of hospitalization, 2 weeks after hospitalization, at discharge, and 6 months after hospitalization. The protocol of this study was registered in CRIS (KCT0003709) and Clinical trial gov (NCT03750591).

#### 2.1.2 Primary objectives

The purpose of this study is to prospectively evaluate the efficacy and safety of integrative Korean medicine treatment that uses acupuncture, chuna manual therapy, pharmacopuncture, and herbal medicine on LDH patients.

#### 2.1.3 Secondary objectives

- To evaluate the clinical progress of LDH with or without spinal stenosis
- To analyze the factors (age, disease level, concomitant disease, and so on) that affect the improvement of LDH patients who received integrative Korean medicine treatment
- To conduct safety evaluation of acupuncture, chuna therapy, pharmacopuncture, and herbal medicine treatment used in the hospital
- To observe the effect of combined use of drugs in patients taking medications for existing conditions (eg, hypertension, diabetes)
- To collect natural history-related data upon Korean medicine treatment
- To determine the quality of life of patients with spine-related diseases undergoing integrative Korean medicine treatment for inpatients in Korea.

### 2.2 Participating centers

The subjects will be recruited from 4 spine specialized Korean medical hospitals. To reflect the regional diversity, the study will be conducted in 4 Korean medical hospitals that are distant from each other. All participating centers are spine specialized Korean medical hospitals designated by the Ministry of Health and Welfare, where integrative Korean medicine treatment using acupuncture, herbal medicine, chuna therapy, and pharmacopuncture is performed. Additionally, as doctors practicing Western medicine conduct consultations there, conservative treatments such as injection and physical therapy can be performed.

### 2.3 Institutional review board approval

All participating institutes in clinical research have been approved by the institutional review board (IRB). The IRB number of each hospital is as follows: Jaseng Hospital of Korean Medicine (JASENG 2017-12-005-002), Jaseng Hospital of Korean Medicine Daejeon Branch (JASENG 2018-02-002), Jaseng Hospital of Korean Medicine Bucheon Branch (JASENG 2018-02-004), and Jaseng Hospital of Korean medicine Haeundae Branch (JASENG 2018-02-006).

### 2.4 Patient enrollment

All patients admitted for LDH treatment at 4 spine specialized Korean medical hospitals will be encouraged to participate, and upon providing them with the study description, written consent will be collected from those that agree to participate.

### 2.5 Subject eligibility

#### 2.5.1 Inclusion criteria

1. Patients confirmed to have LDH by doctors or doctors of Korean Medicine through magnetic resonance imaging (MRI) within the last 3 years
2. Patients with radiating pain (those that complain of radiating pain in any one of the lower extremities)
3. Patients whose intensity of back or radiating pain is a numeric rating scale (NRS) ≥5
4. Patients aged between 19 and 70 years
5. Those who have agreed to participate in the clinical research and have signed the consent form
6. Patients admitted to the Korean medical hospital for treatment

#### 2.5.2 Exclusion criteria

1) Patients who have been diagnosed with certain serious diseases that may cause back pain or neck pain (tumor metastases to the spine, acute fractures, spinal dislocations, and so on)
2) Patients hospitalized due to pain caused by traffic accident
3) Patients who have progressive neurological deficits or severe neurological symptoms such as spinal cord injury
4) Patients with serious mental illness
5) Patients who cannot consent
6) Patients deemed unable to participate in the clinical research by other researchers
7) Patients who were diagnosed with lumbar dislocation higher than Meyerding grade II by doctors or Korean medical doctors

### 2.6 Data collection

#### 2.6.1 Screening process

Participants will be screened using the inclusion/exclusion criteria. MRI imaging and analysis will be performed to confirm LDH. The pain level needs to be checked for the presence of radiating pain per inclusion criteria. Only MRIs taken within the last 3 years will be accepted, and only after confirming that MRI and lower extremity radiating pain meets inclusion criteria will the patients be enrolled in the study. The type and the location of intervertebral herniation in the lumbar spine will also be recorded.

- Whether or not desiccated disc change is accompanied in each level and whether bulging disc is accompanied
- Whether or not extrusion, protrusion, bulging, sequestration, and migration is accompanied in each level
- In case of extrusion or protrusion, confirm the following: central zone, Lt. subarticular zone, Rt. Subarticular zone,—both subarticular zone, left foraminal zone, Rt. foraminal zone, both foraminal zone, Lt. extraforaminal zone, Rt. extraforaminal zone, and both extraforaminal zone
- In case of sequestration/migration, confirm the following: sequestration, inferior migration, and superior migration

To determine whether the patient has spinal stenosis, MRI will confirm the presence of spinal stenosis and neurogenic claudication. Radiological criteria for spinal stenosis may include those that meet the criteria under MRI or those that have been identified by a radiologist. Additionally, the level and type of stenosis will be recorded. Types of stenosis to be recorded are: central, foraminal, and central and foraminal.

#### 2.6.2 Treatment stage

Level of pain, presence of dysfunction, quality of life, and satisfaction level will be examined when hospitalized, 14 days after hospitalization, and during discharge (Table 1)

**Table 1:**
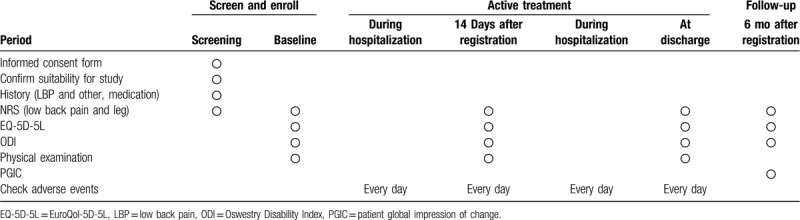
Items to be observed by timepoints.

The items to be surveyed are as follows.

- NRS of waist and legs
- Quality of life: EuroQol-5D-5L (EQ-5D-5L)
- Oswestry Disability Index (ODI)
- Physical examination
- Adverse effects are examined daily by the medical staff during hospitalization
- All records of medical treatments, both western and Korean, performed during hospitalization of the patients

#### 2.6.3 Follow-up

Follow-up assessment will be conducted from the baseline of the treatment to 6 months after the treatment. Follow-up via phone call will assess NRS of waist and legs, EQ-5D-5L, ODI, and Patient Global Impression of Change .

### 2.7 Adverse events

Adverse events (AEs) are undesirable and unintentional signs (ie, abnormal laboratory test result), symptoms, or disease. The researchers will analyze the frequency of AEs, abnormal blood test results, and severe adverse events. During the inpatient treatment period and telephone follow-up, the researchers will confirm and analyze the presence of AEs.

The staff will determine and record the treatments that are likely responsible for the AEs that have occurred and will evaluate the causal relationship between each treatment method and AEs in a six-stage scale (1 = definitely related, 2 = probably related, 3 = possibly related, 4 = probably not related, 5 = definitely not related, and 6 = unknown).

All AEs will be classified into the following 3 levels by Spilker classification^[12]^:

Mild: When no treatment is required and the side effect does not significantly impair the normal life (function) of the subject; Moderate: When the side effect significantly impairs the normal life (function) of the subject, may require treatment, and can recover after treatment, and Severe: When severe adverse reactions require advanced treatment and may have after effects).

### 2.8 Withdrawal and dropout criteria

At any time during the study, the subject may voluntarily withdraw from the study or be dropped out at the researcher's discretion. If the clinical study is discontinued for the subject, the investigator will assess and record the main cause of the dropout.

1) Patients who have been hospitalized for ≤7 days
2) Patients whose guardian or they themselves requests to be withdrawn from the study during the study period or subject withdrawal of consent to participate in the clinical research
3) When progress of the research is deemed unsuitable by other investigators
4) If the patient is not applicable in at least 1 “selection criterion"
5) If the patient is not applicable in at least 1 “exclusion criterion"
6) Subjects whose systematic disease was not found in the pre-clinical examination
7) If the subject cannot participate in follow-up

### 2.9 Data management

An investigator from the coordinating center will distribute a standard operating procedure (SOP) to be used in study processes such as Case Report Form (CRF) completion and data input, and will provide instruction for assessor and investigators at each center. CRF data will use double data entry, and data entry will be performed by each participating center and the coordinating center. After cross-checking the input data, access to the data will be blocked from all investigators except the data coordinator.

### 2.10 Statistical analysis

In this study, intention-to-treat will be the primary analysis. In case of missing values, among multiple imputation, Markov Chain Monte Carlo, and Last observation carried forward will be implemented. Additionally, differences between the baseline and the following timepoints will be tested using paired *t* test or Wilcoxon signed rank test, and differences in time by treatment interactions will be subjected to repeated measures of analysis of variance; if necessary, linear mixed model analysis will also be conducted. Continuous variables are presented as mean (standard deviation) or median (quartile), and categorical variables are expressed in frequency (%) and will be subjected to *χ*^2^ test or Fisher exact test. All statistical analyses were performed by SAS version 9.1.3 statistical package (SAS Institute, Cary, NC) and *P* < .05 was considered statistically significant.

### 2.11 Dissemination

The results of this study will be registered on clinicaltrials.gov, and will be published in an academic paper.

## 3 Discussion

Our study aimed to establish a prospective registry of 1000 patients to overcome the limitations of Korean integrative medicine or CAM-related research, which involved a small number of patients or a large number of retrospective patients, and to confirm the safety and efficacy of Korean medicine treatment for lumbar disc herniation. Additionally, the effect of the treatment was not only observed immediately after the treatment, but also 6 months after, to analyze the long-term treatment effect of Korean medicine treatment for LDH, which is common in recurrence of chronic pain. Also, as a large number of patients were recruited, we can analyze whether the treatment effect differed among CAM inpatient and whether individual characteristics such as age, sex, and presence of stenosis and accompanying diseases influenced the time of improvement. This study was designed as the first project of the Joint and Spine Pain Integrative Evaluation registry (J-SPINE) project, and in the future, we plan to build a registry for other spinal and joint diseases.

However, the limitation of this study is that it will be conducted without a control group. Although the data on safety can be collected in large numbers, those on effectiveness may be limited due to the absence of the comparison group. However, we plan on recruiting outpatient patients or patients receiving nonintegrated Korean medicine treatment through other registry studies. Through this, it will be possible to check whether inpatient Korean medicine treatment is more effective than the outpatient treatment and on which patients it would be more effective towards. Moreover, if the analysis is carried out according to whether there is a difference in the effects depending on the presence of stenosis and whether the treatment was accompanied with Western medical conservative treatment (eg, analgesic drug treatment, electrophysical treatment, and so on), it is possible to analyze the advantages of combination treatment even without the control group. Additionally, we can analyze whether there is a difference in treatment effects according to age or sex.

Another limitation is that 1000 patients may be large in recruitment but lack safety aspects; during hospitalization, systematic investigation will be conducted with Korean medical doctors investigating and recording the side effects on a daily basis, but in terms of safety, it can be a small number to collect rare events. However, if the system is established and the registry research is expanded through this study, it can serve as a foundation to accumulate safety data in the future.

Additionally, it is expected that there will be many dropouts, as the protocol is configured so that many people with <7 days of inpatient treatment will be withdrawn or drop out. In reality, some patients are admitted for examination and treatment just for insurance benefits if private insurance only supports those with hospitalization, and those with severe initial pain and movement restrictions may improve quickly and will be hospitalized and discharged in a short time. In these cases, hospital stays tend to be shorter. Although we tried to establish a registry to properly validate the efficacy of Korean medicine treatment and exclude inpatients from the test, there may be possibilities where the rate of dropouts is high or the information about the late adverse events in dropout patients is missing. We plan on thoroughly reviewing the reasons for dropouts and revisit those patients with adverse effects during hospitalization.

The strength of this study is that it seeks to recruit many patients, as many as 1000. It is also the first study to prospectively recruit those going through CAM inpatient treatment to examine its efficacy and safety in many people. We will not only confirm the safety and efficacy of integrative Korean medicine treatment in 1000 LDH patients but also analyze whether the individual characteristics such as presence of stenosis, sex, age, and type of disc hernia can affect the treatment. This study is expected to not only provide the basis for validating CAM treatment but to also establish the basis for safety, which has been lacking in the past.

## Author contributions

IH and YJL conceptualized and designed the study. JK and SHL managed and coordinated responsibility for the research activity planning and execution. JK, MYK, HWC and MRK conducted a research and investigation process. YJL drafted the article; IH and SHL critically revised the draft. All authors have read and approved the final manuscript.
